# When the Gallbladder Follows The Right Heart: A case report of cardio-biliary syndrome in pulmonary arterial hypertension

**DOI:** 10.1016/j.radcr.2025.09.044

**Published:** 2025-10-10

**Authors:** Mehdi Ayoub Laaroussi, Mohamed El Yamani, Oumaima Ezzahraoui, Imane Drissi, Nadia Fellat, Rokya Fellat

**Affiliations:** a Department of Cardiology, Ibn Sina University Hospital Center, Rabat, Morocco; b Department of Gastrology, Mohammed V Military Instruction Hospital, Rabat, Morocco

**Keywords:** Pulmonary arterial hypertension, Right heart failure, Congestive hepatopathy, Biliary sludge, Cardio-biliary syndrome

## Abstract

Pulmonary arterial hypertension (PAH) is a progressive condition that may lead to chronic right heart failure and systemic venous congestion. One of the lesser-known consequences of this hemodynamic overload is passive hepatic congestion and impaired bile clearance. In rare instances, this pathophysiological process extends to the gallbladder, promoting the formation of sludge and symptoms in the right upper quadrant that may mimic those of primary biliary disease. We report the case of a 42-year-old woman with idiopathic PAH complicated by right heart failure, who presented with persistent abdominal discomfort and vomiting. Imaging revealed congestive hepatopathy and gallbladder sludge without any signs of inflammation or acute cholecystitis. The clinical and imaging findings pointed to a cardio-biliary mechanism. An elective cholecystectomy was performed in the absence of inflammatory signs, leading to complete symptom resolution. This case underlines the importance of recognizing PAH-related hepatobiliary complications and differentiating them from primary biliary pathology, as it significantly impacts management and may prevent unnecessary surgery.

## Introduction

Pulmonary arterial hypertension (PAH) is a chronic and progressive disorder characterized by elevated pulmonary arterial pressures, ultimately leading to right ventricular (RV) failure due to increased afterload on the right heart [1]. As RV dysfunction progresses, the resulting rise in central venous pressure contributes to passive hepatic congestion, which disrupts normal liver architecture and impairs bile flow [2]. While the hepatocardiac axis is well-documented in the context of congestive hepatopathy, its downstream effects on the biliary system remain underrecognized.

Biliary sludge refers to the accumulation of cholesterol crystals, calcium bilirubinate, and mucin within the gallbladder, often considered a precursor to gallstones or cholecystitis [3]. In most cases, biliary sludge is associated with risk factors such as fasting, pregnancy, or parenteral nutrition [3]. However, venous congestion and cholestasis due to right heart failure may also promote sludge formation by altering bile composition and impairing gallbladder motility [2]. This pathophysiological link, though plausible, is rarely emphasized in clinical practice and may lead to misattribution of biliary symptoms to primary gallbladder disease.

This report discusses the diagnostic and therapeutic challenges posed by biliary sludge in a patient with chronic right heart failure due to idiopathic PAH. By examining the cardio-biliary interaction in this context, the case underscores the importance of considering right-sided cardiac dysfunction in the differential diagnosis of hepatobiliary symptoms and encourages clinicians to adopt an integrative approach to abdominal manifestations in this population.

Our paper was written in accordance with the CARE guidelines [4].

## Case report

A 42-year-old woman, with no cardiovascular risk factors and a long-standing history of idiopathic PAH diagnosed 13 years earlier, presented to the cardiology department for evaluation of persistent discomfort in the right upper abdomen, accompanied by intermittent postprandial vomiting. These symptoms had gradually worsened over the past year, without fever, jaundice, anorexia, or weight loss. The patient reported good adherence to her prescribed PAH therapy, which included diuretics and pulmonary vasodilators, with no recent medication changes.

Her past medical history was notable for PAH complicated by right heart failure. She had experienced several prior episodes of peripheral edema and exertional dyspnea, but had remained clinically stable in recent months.

On physical examination, the patient appeared well-oriented and comfortable at rest. Blood pressure was 110/70 mmHg, and heart rate was 78 beats per minute with an irregular rhythm. Cardiovascular auscultation revealed a loud pulmonary component of the second heart sound (accentuated P2) and a systolic murmur consistent with tricuspid regurgitation. Jugular venous distension, hepatomegaly, and bilateral pitting edema of the lower limbs were present, suggestive of right-sided heart failure. Pulmonary auscultation revealed bibasilar crackles. Cutaneous examination noted digital clubbing and inflammatory discoloration of the lower extremities consistent with stasis dermatitis (ochre dermatosis).

Initial laboratory workup revealed a mild cholestatic pattern. Alkaline phosphatase (ALP) was modestly elevated, while total bilirubin was within the upper normal range. Aspartate aminotransferase (AST) and alanine aminotransferase (ALT) were normal, excluding significant hepatocellular injury. Gamma-glutamyl transferase (GGT) was mildly elevated. No evidence of hemolysis, infection, or inflammatory markers was noted. Renal function and coagulation profile were within normal limits.

**Fig. 1:**
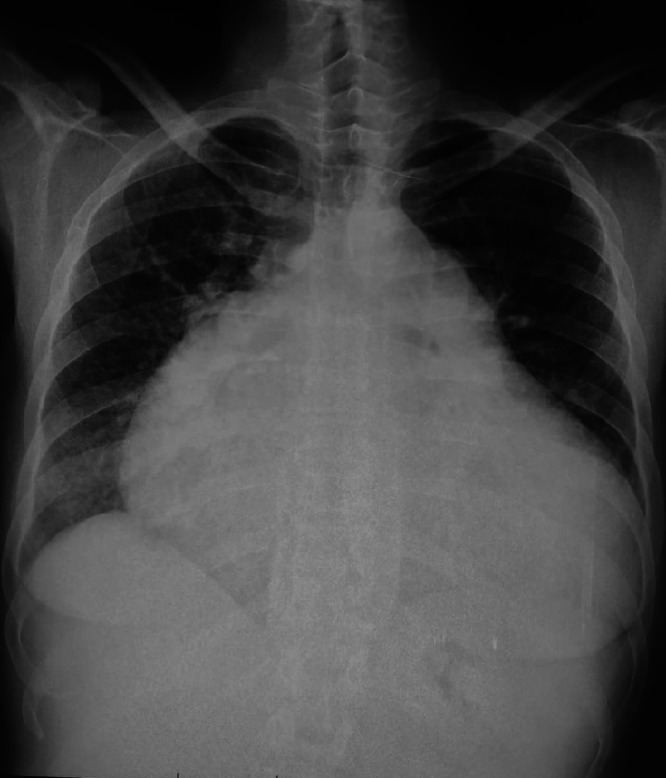
Chest radiography (posteroanterior view) demonstrating cardiomegaly with a prominent right heart border and enlarged main pulmonary artery segment, consistent with chronic pulmonary arterial hypertension.

A chest radiograph demonstrated cardiomegaly with a prominently enlarged pulmonary artery segment and peripheral pulmonary oligemia, consistent with severe pulmonary hypertension. These radiographic findings supported the suspicion of chronic RV pressure overload (Fig. 1)

Transthoracic echocardiography revealed a structurally normal left ventricle with preserved systolic function (LVEF 56%). In contrast, the right ventricle was severely dilated (basal diameter 57 mm) and hypokinetic, with reduced systolic function (tricuspid annular systolic velocity at 6 cm/s). The right atrium was markedly enlarged (surface area 52 cm²), and the pulmonary artery trunk measured 27 mm in diameter, further confirming the hemodynamic burden of PAH.

**Fig. 2:**
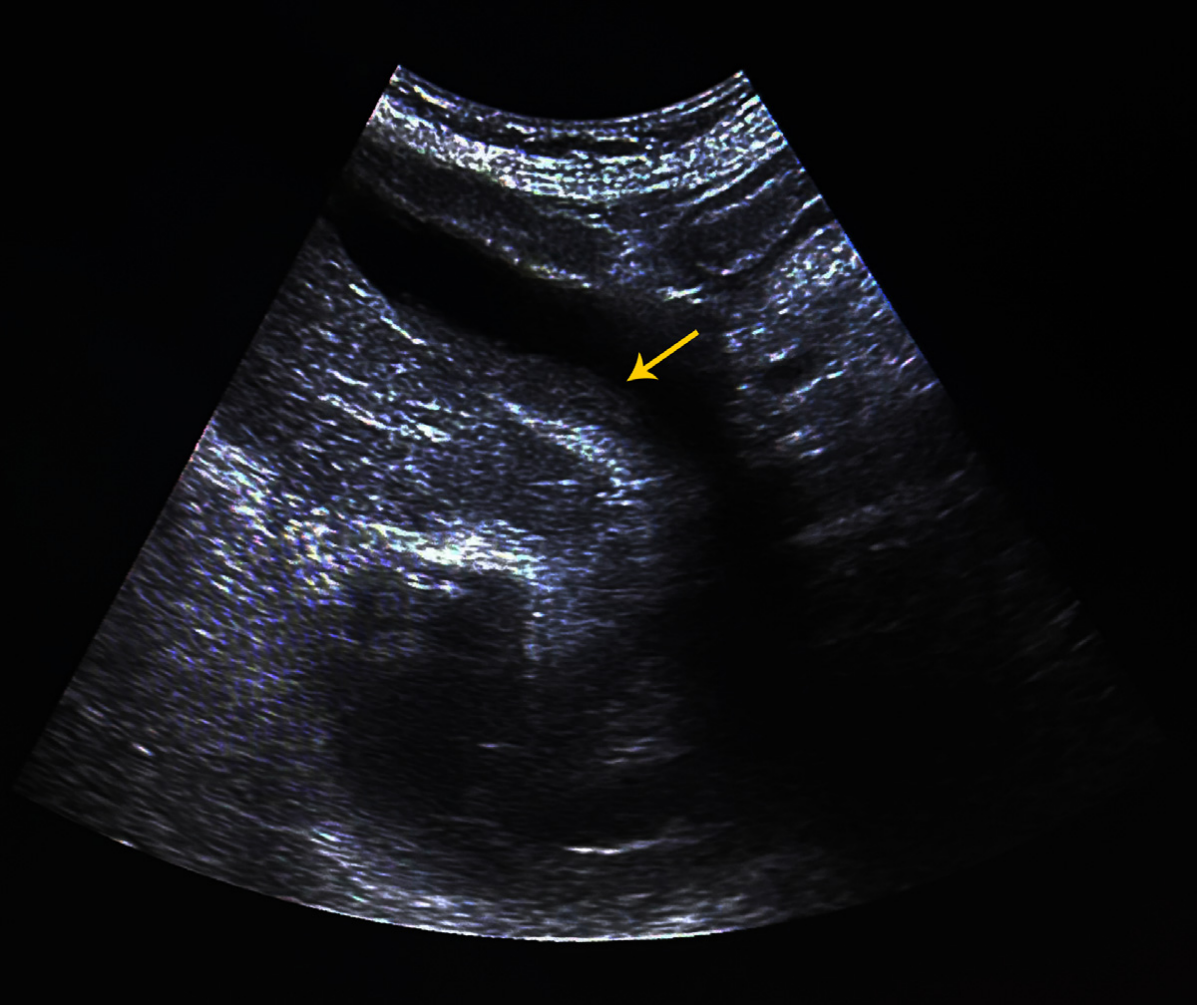
Abdominal ultrasound showing a longitudinal view of the dysmorphic gallbladder. A fine, hyperechogenic, non-shadowing material is seen layering dependently within the lumen (yellow arrow), consistent with biliary sludge.

Abdominal ultrasonography showed a dysmorphic, hypoechoic liver with significant hepatic vein dilatation — a pattern suggestive of passive hepatic congestion. The gallbladder was dysmorphic and contained an aggregation of fine microlithiasis (biliary sludge) without evidence of wall thickening, pericholecystic fluid, or intrahepatic ductal dilatation (Fig. 2). No biliary obstruction or inflammatory signs were noted.

Given the chronicity of her symptoms, radiologic confirmation of biliary sludge, and associated right heart dysfunction with hepatic congestion, the patient underwent an elective laparoscopic cholecystectomy. Intraoperative findings were unremarkable, and no complications occurred. Her postoperative course was favorable, with complete resolution of RUQ pain and vomiting within days.

## Discussion

**Fig. 3:**
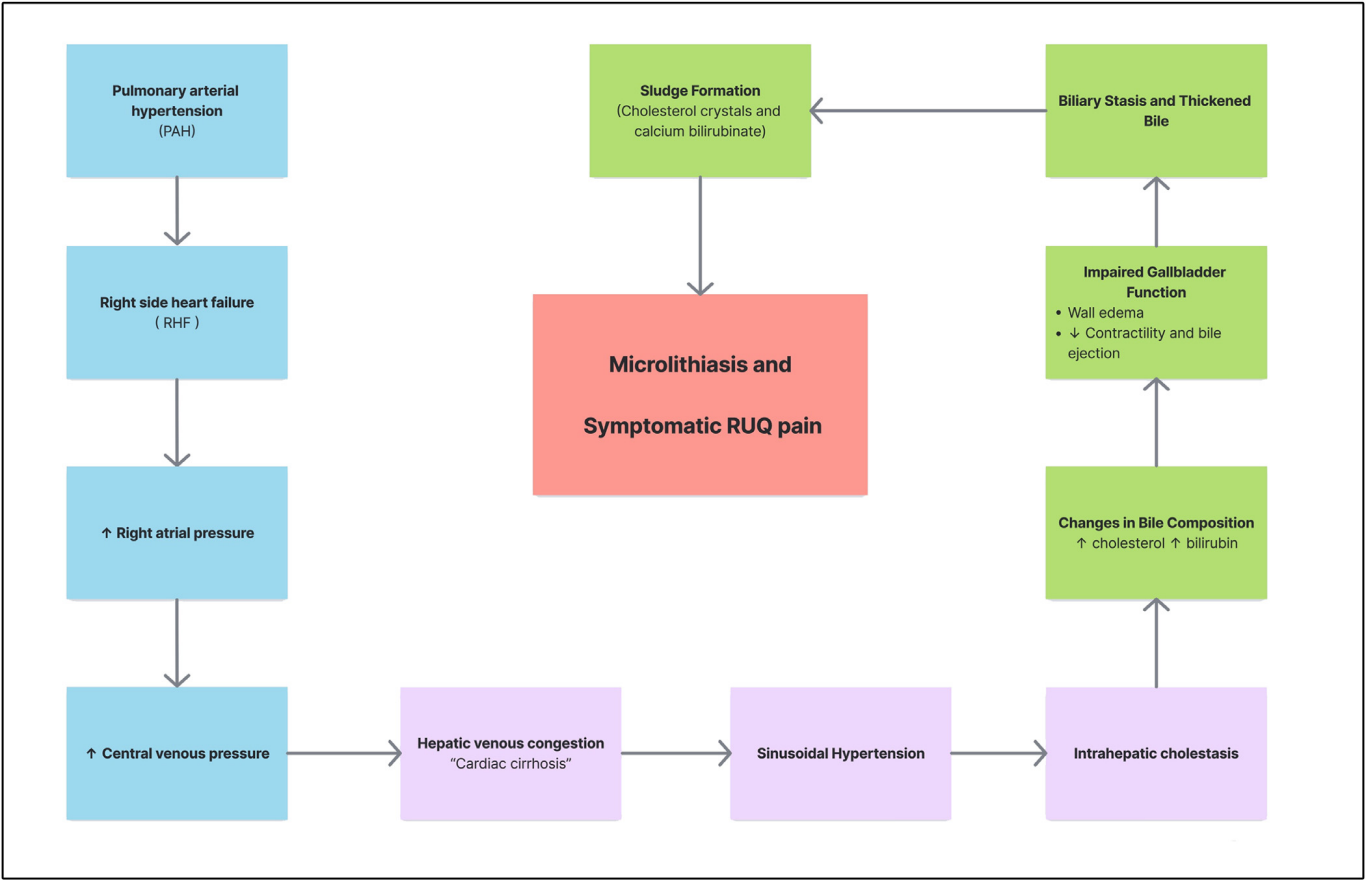
Schematic representation of the pathophysiological cascade linking pulmonary arterial hypertension (PAH) to hepatic congestion, bile stasis, and biliary sludge formation. PAH leads to right-sided heart failure and elevated central venous pressure, resulting in hepatic venous congestion and intrahepatic cholestasis. These changes alter bile composition and impair gallbladder function, promoting biliary stasis and sludge formation. The accumulation of cholesterol crystals and calcium bilirubinate contributes to microlithiasis, ultimately causing RUQ pain in the absence of acute inflammation.

Chronic right-sided heart failure (RHF) is defined as the sustained inability of the right ventricle to generate adequate forward flow into the pulmonary circulation, resulting in elevated systemic venous pressures and tissue congestion [5]. In the context of advanced PAH, chronic pressure overload progressively impairs RV systolic function, ultimately raising right atrial and central venous pressures [6,7]. This backward transmission of pressure extends to the hepatic veins and sinusoids, giving rise to passive hepatic congestion—a condition often referred to as “cardiac cirrhosis” [6]. Chronic venous congestion has deleterious effects on liver architecture. Specifically, increased sinusoidal pressure leads to centrilobular sinusoidal dilation, hepatocyte atrophy, and pericentral fibrosis, which collectively compromise the liver’s metabolic and excretory functions [6]. Among the most affected pathways is bile excretion: elevated hydrostatic pressure within the sinusoids disrupts hepatocellular tight junctions and the integrity of bile canaliculi, thereby impairing bile flow and facilitating leakage of bile constituents into the hepatic interstitium [2]. As a consequence, longstanding venous congestion promotes both intrahepatic cholestasis and structural remodeling of the biliary tree [8]. This includes canalicular dysfunction, biliary metaplasia, and ductular proliferation. Notably, these changes often occur in the absence of extrahepatic obstruction and are reflected in a predominantly cholestatic pattern of liver enzyme elevation—most commonly increased levels of bilirubin, alkaline phosphatase (ALP), and gamma-glutamyl transferase (GGT) [8]. This same pathophysiological process extends to the gallbladder, promoting biliary stasis through multiple mechanisms. Elevated venous pressures within the portal and cystic veins lead to gallbladder wall edema and impaired contractility, thereby reducing bile ejection [9]. Concurrently, bile becomes increasingly saturated with cholesterol and bilirubin due to intrahepatic cholestasis, predisposing it to precipitation [10]. In low-flow states—such as advanced heart failure, dehydration, or prolonged fasting—bile stagnation intensifies, and insoluble components such as cholesterol crystals and calcium bilirubinate may coalesce into sludge [[2], [3], [4], [5], [6], [7], [8], [9]]. Over time, this combination of impaired motility and altered bile composition creates an environment favorable to microlith formation. This cardio-hepato-biliary pathophysiological sequence is summarized in Fig. 3.

Clinically, manifestations of congestive hepatopathy such as hepatomegaly, mild jaundice, and right upper quadrant discomfort are believed to result not only from hepatic capsular distension but also from associated biliary stasis [[7], [8], [9], [10], [11]]. Autopsy and histopathologic studies in patients with chronic RV failure, including those with PAH, have demonstrated sinusoidal dilation, canalicular rupture, and extravasation of bile pigments into the hepatic parenchyma, supporting a mechanistic link between venous congestion and bile flow disruption. Thus, in the setting of chronic right heart failure, the congested liver contributes to both systemic cholestasis and functional impairment of gallbladder drainage, ultimately facilitating the accumulation of biliary sludge [2].

Despite this plausible pathophysiology, cardio‐biliary sludge is rarely described in the literature. Most prior reports focus on acute acalculous cholecystitis (AAC) in critically ill or shock patients, or on gallbladder wall edema in HF, rather than sludge formation. A few case reports and series hint at the phenomenon. For example, Desautels et al. described a patient with decompensated CHF who's transient “cholecystalgia” (RUQ pain with gallbladder edema) mimicked biliary colic; initial workup even considered cholecystectomy before HF therapy clarified the cause [12]. Similarly, Habib et al. reported HF patients with acute RUQ pain whose imaging showed gallbladder wall thickening without stones, findings later attributed to volume overload rather than true cholecystitis [11]. In these case-based discussions, CHF was recognized to cause congestive hepatopathy and isolated gallbladder edema (“cholecystalgia”). Yu et al. note that diffuse gallbladder wall thickening on ultrasound is often due to systemic causes like CHF [9]. Larger studies are sparse. A recent series of 18 cardiac patients with presumed acute cholecystitis found gallbladder sludge (or stones) in 50% by ultrasound [13], but these were hospitalized patients, often with ischemic or septic triggers, and not a focus on idiopathic RHF. To our knowledge, no cohort study has explicitly linked chronic PAH/HF with gallbladder sludge as a primary finding. Likewise, reviews of congestive hepatopathy discuss sinusoidal damage and cholestasis, but do not comment on gallbladder sludge [7]. In summary, although congestive hepatopathy is well‐recognized, its direct impact on gallbladder bile (stasis/sludge) is rarely addressed. The handful of documented cardio‐biliary cases underscores how easily biliary manifestations can be overlooked.

This case underscores the diagnostic challenge of interpreting right upper quadrant (RUQ) pain and biliary sludge in patients with chronic right heart failure. Such findings are often misattributed to primary gallbladder pathology, including microlithiasis or early cholecystitis. However, in individuals with advanced PAH and systemic venous congestion, the etiology may be cardiogenic [14]. Laboratory evaluations can aid in differentiation. Congestive hepatopathy often results in mild to moderate elevations of cholestatic enzymes, such as alkaline phosphatase (ALP) and bilirubin, without significant hepatocellular injury [15]. Imaging findings, while non-specific, should be interpreted within the clinical context. Gallbladder wall thickening and sludge can be seen in heart failure without indicating primary biliary pathology. The absence of pericholecystic fluid, gallstones, or a positive sonographic Murphy's sign further supports a non-inflammatory etiology [16]. A similar diagnostic dilemma was described by Guarino et al., [17] who reported a case of hepatic inflammatory pseudotumor (IPT) presenting with RUQ pain and contrast-enhanced ultrasound findings suggestive of malignancy. The lesion ultimately resolved spontaneously and was identified as a benign inflammatory process. This scenario is analogous to the present case, in which gallbladder sludge in a patient with chronic right heart failure could be misinterpreted as primary biliary disease. Both cases underscore the crucial importance of interpreting imaging findings within the broader clinical and hemodynamic context to prevent misdiagnosis and unnecessary interventions.

The distinction between congestive hepatopathy and primary biliary disease has immediate management implications. Recognizing gallbladder edema and biliary sludge as manifestations of congestive hepatopathy directs therapy towards optimizing cardiac function. Diuresis and management of heart failure or PAH reduce hepatic venous pressure, often alleviating right upper quadrant symptoms [12]. Similarly, Shamban et al. [18] documented that aggressive diuretic therapy markedly lowered alkaline phosphatase levels in their heart failure patient with biliary sludge. Habib et al. also noted that gallbladder edema resulting from heart failure may resolve with improved cardiac function [11]. Conversely, surgical intervention, such as cholecystectomy, carries a high risk in decompensated heart failure and may not relieve symptoms if underlying congestion persists. Case reports warn that heart failure-related gallbladder findings often led to plans for cholecystectomy until the cardiac origin was recognized [12]. In 1 series, ultrasonographic gallbladder wall thickening in heart failure was initially misinterpreted as cholecystitis, only for the patient to rapidly improve with volume management [19]. Thus, failing to appreciate the cardio-biliary link can lead to unnecessary surgery or delay in treating the true problem.

To conclude**,** abdominal symptoms in the setting of systemic venous congestion should prompt a holistic evaluation that considers the interconnected roles of the heart, liver, and biliary system. Rather than approaching hepatobiliary abnormalities in isolation, clinicians should maintain a high index of suspicion for functional biliary involvement secondary to cardiac dysfunction. A multidisciplinary perspective, including cardiology, hepatology, and radiology, can help clarify the underlying mechanism and support more targeted, non-invasive management strategies.

## Teaching point

The correlation between chronic right heart failure and biliary sludge reflects a complex pathophysiological interplay involving hepatic congestion, impaired bile flow, and gallbladder stasis. This case reinforces the importance of recognizing congestive hepatopathy and its biliary manifestations as potential mimics of primary gallbladder disease. In patients with PAH or decompensated right heart failure, RUQ pain and biliary sludge should prompt consideration of a cardiogenic etiology. Early identification may avoid unnecessary surgical intervention and guide appropriate management through cardiac optimization. Further studies are warranted to elucidate this underrecognized cardio-biliary syndrome's prevalence, diagnostic markers, and clinical outcomes.

## Patient consent

Complete written informed consent was obtained from the patient for the publication of this study and accompanying images.
